# A Preliminary Investigation of a Conceptual Framework of Performance Enhancement Across Different Life Domains

**DOI:** 10.3390/sports13120434

**Published:** 2025-12-04

**Authors:** Lida Skoufa, Despoina Ourda, Vassilis Barkoukis, Haralambos Tsorbatzoudis

**Affiliations:** 1 Department of Physical Education and Sport Science, Aristotle University of Thessaloniki, 57001 Thessaloniki, Greece; lida.skoufa@yahoo.gr (L.S.); despoino@phed.auth.gr (D.O.);; 2 Department of Life and Health Sciences, Frederick University, 1036 Nicosia, Cyprus

**Keywords:** doping, neuroenhancement, performance enhancement, goal system theory

## Abstract

The use of chemically assisted performance enhancement (CAPE) substances has become a prominent trend in today’s competitive societies. Although evidence suggests that CAPE behaviors across different life domains share common characteristics, no consistent theoretical framework exists for understanding the decision to engage in such behaviors. The aim of the present study was to examine a unified conceptualization of CAPE behaviors in four life domains. A total of 254 participants (64 competitive athletes, 40 recreational exercisers, 67 students, and 83 professionals) completed a survey assessing distal and proximal associations of CAPE behaviors (adapted for each domain). Path analysis used to evaluate the proposed model demonstrated an adequate fit. Results indicated that proximal associations of intentions (i.e., attitudes, norms, and situational temptation) were predicted by distal variables (i.e., social norms and cultural values). Intentions to engage in CAPE behaviors were predicted by attitudes and situational temptation. Goal commitment predicted only the mean of working, studying, or training. Finally, the mean of supplement use was predicted by norms. These findings provide preliminary evidence for a conceptual framework to understand CAPE behaviors across life domains, which may serve as a basis for designing intervention programs aimed at helping individuals make informed decisions about CAPE.

## 1. Introduction

A widespread phenomenon in modern lifestyle is the chemically assisted performance enhancement (CAPE), defined as the use of drugs for non-medical purposes. In an attempt to pursue excellence, individuals from various disciplines, including students, academics, athletes, and recreational exercisers, use drugs to enhance their intelligence, abilities, performance, and fitness [1,2]. Two main categories of CAPE have been identified: the use of drugs to improve physical performance and appearance and the use of drugs for cognitive enhancement (neuroenhancement).

CAPE behaviors are found in both physical and mental areas, with people in many fields using these methods to boost their abilities or gain a competitive edge. With respect to physical performance, CAPE refers to the use of doping substances (e.g., anabolic steroids), dietary supplements, and other over-the-counter products by athletes or other professionals (e.g., military officers) to enhance their physical performance [3,4,5]. Moreover, recreational exercisers use these substances for appearance purposes, such as increasing muscle mass and reducing body fat [6,7]. For cognitive enhancement, CAPE includes drugs (e.g., methylphenidate, modafinil) used by students, academics, and professionals (e.g., doctors) to improve concentration, manage stress, and reduce sleep needs [8,9]. While doping in sport and neuroenhancement in academic or professional contexts differ in their social meaning and regulatory status, both can be conceptualized as manifestations of CAPE behaviors that share common psychological mechanisms.

Regarding prevalence, sport and exercise are the most commonly studied CAPE behaviors. Although accurately estimating the prevalence of doping in sports is challenging, research indicates that between 14% and 39% of competitive athletes have experience with doping substances, depending on the sport, country, and competitive level [4,7]. However, using the randomized response technique, Ulrich et al. [10] found that the prevalence could be as high as 57.1% in elite competitions. In recreational sports, evidence suggests that 19.3% of exercisers have had some experience with doping substances [7], while 15% to 26% of non-users are considering or are curious about the effects of these substances [11,12]. Furthermore, military personnel and police officers are also at risk of using such substances [13,14]. A study of personnel in the US Navy and Marine Corps revealed that 73% of participants regularly used nutritional supplements [15]. The prevalence rates of neuroenhancement are similarly high and might even be underestimated [16]. Estimates suggest 6.9–55% of college students, 4% of school children, 9.73% of teachers, 19% of professionals in economics, 8.9% of surgeons, and 4.2% of the general population misuse drugs to improve cognitive function [8,17,18,19,20,21]. However, it has been suggested that prevalence may vary across different cultures [21].

Despite the commonalities in CAPE behaviors across different contexts, existing literature lacks a unified conceptual understanding of the psychological mechanisms that lead individuals to engage in these behaviors. The present study aims to address this gap by providing preliminary evidence for a model that unifies and extends existing conceptualizations of CAPE behaviors across sport and lifestyle domains.

Previous research has frequently employed the Theory of Planned Behavior (TPB) [22] to examine performance enhancement intentions, showing that attitudes (i.e., evaluations of the behavior), subjective norms (i.e., perceived social pressure), and perceived behavioral control (i.e., perceived ability to perform the behavior) are consistent predictors in both competitive and recreational sport [23,24,25]. Extensions of this work have also applied the TPB framework to neuroenhancement, where past behavior, attitudes, subjective norms, and perceived ease of access were found to predict intentions to use substances such as caffeine tablets or prescription stimulants [26,27,28,29,30]. While this evidence highlights the utility of TPB across different CAPE contexts, the model has been criticized for its limited scope, as it does not account for distal or background factors that indirectly shape intentions. The Integrative Model of Behavioral Prediction (IMBP) [31] addresses this limitation by incorporating distal variables (e.g., beliefs, achievement goals, situational temptation, anticipated regret, descriptive norms) and background characteristics, which indirectly influence attitudes, subjective norms, and perceived behavioral control. Studies in competitive sport have confirmed the utility of this extended framework [32,33]. Research has shown that personality traits such as sensation seeking and risk perception play an important role in doping and related CAPE behaviors. Higher levels of sensation seeking have been linked with greater willingness to experiment with performance-enhancing substances, while lower perceptions of risk are associated with stronger performance enhancement intentions [34,35,36]. These findings suggest that psychological dispositions interact with social-cognitive predictors to shape engagement in CAPE, and they can be conceptualized within the IMBP [31] as distal or background variables indirectly influencing attitudes, subjective norms, and perceived behavioral control. CAPE behaviors in sport and other domains share structural similarities suggesting findings from one domain could apply to another [37]. Therefore, although the IMBP has not yet been applied in neuroenhancement, it can be assumed to effectively describe the decision-making process in this context as well. Evidence has confirmed the effect of distal variables on proximal predictors (e.g., attitudes) of CAPE behaviors. For instance, individuals who perceive these drugs as harmless, feel confident about their knowledge of safe usage, are more competitive and have surface motives hold more positive attitudes toward their use [38,39]. Similarly, perceived unfairness and academic performance have been linked to more negative attitudes [38,39].

Although the IMBP has been shown to effectively predict CAPE intentions and behaviors, it suggests that it represents the end of a decision-making process. However, Petróczi [40] proposed that doping serves as a means for performance enhancement, while Wolff and Brand [37] argued that the use of enhancers is a means to achieve significant ends in both sports and everyday life (e.g., winning a medal or succeeding academically). Tsorbatzoudis et al. [41] further advocated that doping is a means to an end rather than an end in itself. It is reasonable to assume that performance enhancement is not the ultimate reason for engaging in CAPE behaviors. Instead, performance enhancement is a necessary step for individuals to achieve higher-order goals (e.g., money, fame) [42]. In this sense, CAPE behaviors can be viewed as steps toward achieving an individual’s higher goals through performance enhancement. Although this process has not yet been examined in the sport literature, the Goal Systems Theory [43] could serve as a robust theoretical framework to support this hypothesis.

The Goal Systems Theory is a motivational cognitive approach suggesting that goal systems comprise mental networks where goals are cognitively linked to means of attainment, while means are also connected to other means [43]. According to the theory, multiple means may be associated with a particular goal; for example, proper diet, adequate sleep, and nutritional supplements can help an athlete achieve the ultimate goal of participating in the Olympic Games. Furthermore, means are connected with other means; proper diet, sufficient sleep, and nutritional supplements improve athletes’ performance, which is an essential means to achieving the ultimate goal of Olympic participation. Additionally, multiple goals might be linked to a single means; for instance, performance enhancement is expected to result in participation in the Olympic Games and securing better contracts. The strength of the cognitive links between goals and means is determined by their number; when the number of means is small, the link between them is stronger. The theory also emphasizes goal commitment and goal difficulty as crucial factors influencing the quality of implementing the chosen means ultimately affecting the achievement of the pursued goals [43]. According to the theory, the strength of an individual’s intention toward a behavior, e.g., doping, depends on both the perceived value of the goal and the perceived instrumentality of the means. When a means (e.g., a specific behavior) is strongly associated with a valued goal, individuals are more likely to form strong intentions to engage in that behavior. Moreover, goal difficulty and commitment play critical roles: difficult but attainable goals tend to enhance commitment, which in turn increases the likelihood of pursuing relevant means. Thus, the alignment between means, goal value, and personal commitment determines the motivational strength underlying behavioral intentions [43].

### The Present Study

Existing evidence suggests that CAPE behaviors across different sport and lifestyle contexts share several commonalities [37]. For instance, common theoretical approaches have been tested in various life domains, such as sports, exercise and academia. However, no studies have yet adopted a unified theoretical framework to understand the psychological processes underlying the decision to engage in these behaviors. The present study aims to preliminarily test the integration of these approaches into a common theoretical framework suitable for describing the underlying processes leading to CAPE behaviors across four life domains: sport, exercise, studentship, and work. Although dietary supplements, prescription drugs, and illicit doping substances differ considerably in terms of accessibility, legal status, and associated health risks, they were included under the same conceptual framework to capture the full spectrum of performance-enhancing practices [15,44,45]. This inclusive approach enables the examination of common psychological mechanisms, while recognizing that the severity, motivations, and consequences of such behaviors differ across categories. Furthermore, the present study seeks to expand existing conceptualizations of CAPE behaviors. Previous research has treated CAPE behaviors as the end of the psychological process primarily focusing on identifying their determinants. Although some scholars argue that CAPE behaviors should not be viewed as end states but rather as part of a broader process that helps individuals achieve their ultimate goals [37,40,41], there is still a lack of systematic evidence and a solid conceptualization of this process. This study aims to preliminarily investigate this hypothesis based on the Goal Systems Theory [43].

Based on existing evidence, we formulated the following hypotheses:

**H1.**  *Individual traits (i.e., perfectionism, cultural values, self-determination, group identification and orientation, distal norms, and past behavior) will be associated with proximal correlates of CAPE behaviors (i.e., attitudes, proximal norms, perceived behavioral control, and situational temptation) [32].*

**H2.**  *Proximal correlates of CAPE behaviors will be associated with intentions toward adopting CAPE behaviors and means [32,46].*

**H3.**  *Drawing from Goal Systems Theory, goal commitment and goal difficulty will be associated with the use of means [40,41,43].*

**Figure 1:**
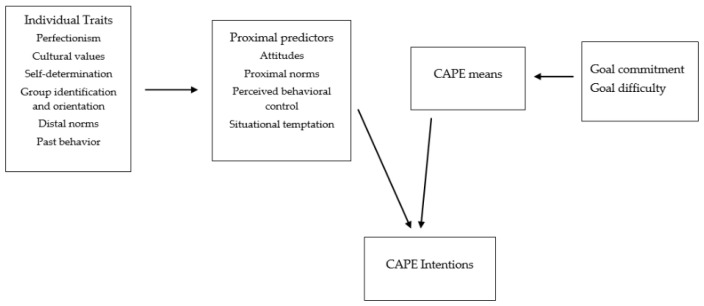
Proposed conceptual framework of CAPE behaviors.

**H4.**  *The use of means will, in turn, be associated with intentions toward CAPE behaviors [37,43] (see Figure 1).*

## 2. Materials and Methods

### 2.1. Participants

Participants were 254 individuals from Greece (Mage = 28.92, SD = 10.85; 113 males). Of these, 64 were competitive athletes (Mage = 19.92, SD = 3.50; 30 males), 40 were recreational exercisers (Mage = 25.60, SD = 11.49; 10 males), 67 were students (Mage = 25.48, SD = 8.98; 22 males), and 83 were professionals (Mage = 44.71, SD = 11.12; 51 males).

The inclusion criteria for each subgroup were informed by previous literature [7] and adapted to the aims of the present study. Participants were categorized as competitive or recreational based on their self-reported level of organized sport involvement. Competitive athletes were defined as individuals who regularly participated in officially sanctioned competitions and followed a structured training program guided by a coach or club. Recreational exercisers referred to individuals who engaged in exercise or sport primarily for leisure, fitness, or personal enjoyment, without systematic participation in competitions. Both groups were required to train systematically for at least three times per week. Students were recruited from faculties with high academic workload (e.g., medicine, engineering) [8]. Professionals considered at risk for CAPE behaviors (e.g., academics, policy makers, army officers) [5] were also targeted.

Recruitment was conducted online through social media announcements, mailing lists of universities and professional institutions, and posts in regional sport clubs and gyms. The final sample size was comparable to previous studies in the field and considered adequate for path analysis [47].

### 2.2. Measures

Means to achieve goals: Participants rated on a 7-point scale the frequency through which they used six means (e.g., proper training, proper diet and nutrition, good sleep, nutritional supplements, performance enhancement substances (PES), etc., in order to achieve their ultimate goal.

Goal commitment and goal difficulty: One item was used to assess goal commitment (“Doing good at ‘work/study/sport/exercise is important to me”) with responses anchored on 10-point scale from 0 (Not important at all) to 100 (Extremely important). One item was used to measure goal difficulty (“Succeeding at ‘work/study/sport/exercise’ is difficult”) rated on a 7-point scale ranging from 1 (Do not agree at all) to 7 (Extremely agree).

PES intentions: Intentions to use PES was measured via six items following the stem question I would use a PED if… (e.g., “…my performance was lower than I expected”). Responses are given on a 7-point Likert-type scale from “strongly agree” (7) to “strongly disagree” (1).

PES behavior: One item was used to measure past PES behavior (“Have you ever used PES to enhance your performance?”). Four different response options were provided (1 = no, I have never used PES; 2 = yes, I once used PES to enhance my performance, but not ever since; 3 = yes, I occasionally use PES to enhance my performance; and 4 = yes, I systematically use PES to enhance my performance).

Perfectionism: The High Standards subscale of The Greek version of the Almost Perfect Scale-Revised (APS-R) [48] was used to measure perfectionism. The subscale includes 7 items (e.g., I expect the best from myself) assessing the high standards one sets for oneself and it is scored on a five-point Likert scale ranging from 1 (strongly disagree) to 5 (strongly agree).

Group-identification and orientation: Three items adjusted from Norman et al. [49] were used to assess self-identity (e.g., “I have a strong identity with my coworkers/teammates/co-exercisers”). Responses were anchored on a 7-point Likert scale, 1 (strongly disagree/not at all), 7 (strongly agree/very much). Group orientation was assessed via two items (e.g., “It is important to me to be in harmony with people in my work/school/team/gym”) [50] with responses coded on a 7-point Likert scale ranging from 1 (strongly disagree) to 7 (strongly agree).

Values: The Gelfand et al. [51] scale was used to measure cultural values through six items (e.g., ‘There are many social norms that people are supposed to abide by in my country’). Responses were provided on a 6-point Likert scale ranging from 1 (strongly disagree) to 6 (strongly agree).

Self-determination: The Self-Regulation Questionnaire was used to assess self-determination. The scales included 15 items measuring intrinsic motivation (e.g., ‘For the pleasure of discovering and mastering new training techniques’), identified regulation (e.g., ‘Because I have a strong value for being active and healthy’), introjected regulation (e.g., ‘Because I feel pressured to work out’), and external regulation (e.g., ‘Because I want others to see me as physically fit’). The items were appropriately adjusted for each life domain. Participants responded on a 7-point Likert scale ranging from not at all true (1) to very true (7).

Motivation to comply: A single item was used to assess motivation to comply (“When it comes to matters of performance enhancement, I want to do what people important to me approve”). The response options ranged from completely disagree (1) to completely agree (7).

Knowledge: PES knowledge was measured with four items (e.g., ‘How often have you looked up for information about PES from an expert?’) and participants responding on a 7-point Likert scale ranging from 1 (not at all) to 7 (very often).

Perceived behavioral control: Two items were used to assess perceived behavioral control (e.g., “I feel in complete control over whether I will use PES to enhance my performance during this season”) measured on a 7-point Likert-type scale ranging from 1 (no control) to 7 (complete control).

Subjective norms: Distal-level subjective norms were measured with two items (e.g., ‘Do you think that the society approves of PES use in work/sports/school/gym’). Proximal-level subjective norms were measured with 6 items (e.g., Do your coworkers/classmates/teammates/co-exercisers approve of substance use for performance-enhancement reasons?’). Responses to these items were assessed on 7-point Likert scales (e.g., 1 = strongly disagree/definitely not/never, 7 = strongly agree/definitely yes/very frequently).

Descriptive norms: Distal level descriptive norms were measured with two items. The first item assessed the use of CAPE by professionals in other work environments/students from other departments/athletes from other sports/exercisers in other exercise settings (e.g., “How many athletes in the same competitive level as you do you believe that use substances to improve their performance?), scored on a 7-point scale from 1 (nobody) to 7 (everyone). The second item assessed the perceived percentage of CAPE used by athletes/exercisers/professionals/students (e.g., ‘Out of 100%, how many athletes/exercisers/professionals/students in your country do you think engage in substance use to enhance their performance?’). Responses ranged from 0 to 100%. Proximal-level descriptive norms were assessed with one item (e.g., How many of your coworkers/classmates/teammates/co-exercisers do you think engage in PES use to enhance their performance’). Also, items about social-moral atmosphere (e.g., ‘How many of your coworkers/classmates/teammates/co-exercisers would engage in PES use, if it was necessary for them to enhance their performance?’) [52]) were used as an additional measure of proximal descriptive norms. Lastly, the frequency of exposure to PES incidents was assessed with two items (e.g., ‘In the last year, how often have you heard about exercisers/athletes/students/professionals engaging in PES use’). In all these items participants responded on a 7-point continuous scale (1 = lower end, 7 = higher end).

Attitudes: Attitudes were measured with eight 7-point bipolar adjectives (e.g., bad-good, harmful-beneficial, ethical-unethical, and useful-useless): following the stem question “The use of PES to enhance my performance during this season is…”

Situational temptation: Efficacy to resist temptation to use PES was assessed with the question “How sure you are that you would be able to resist temptation to use performance enhancement substances if…” followed by seven items (e.g., ‘My performance was much worse than expected”). Responses were anchored on a 5-point Likert scale (1 = not at all tempted, 5 = very much).

### 2.3. Procedure

The study received approval from the respective committee of the authors’ university. The study aims and survey were communicated to stakeholders (i.e., club directors and coaches, gym owners and instructors, rector and dean authorities, police and army officials) in order to obtain permission to distribute the survey. Four online surveys were developed, one for each life domain. Following obtaining approval, the links to the online survey were sent to participants. The link included a sheet providing information on the aims and objectives of the study, the anonymity, the confidentiality of their responses, the use of their response for research purposes solely, GDPR guidelines and contact persons. Following the information sheet participants were presented with an informed consent form. Only participants providing inform consent took part in the study.

### 2.4. Data Analysis

Descriptive statistics and correlation coefficients were performed with SPSS version 25.0. The study’s hypotheses were tested via path analysis. Due to the complexity of the model, the large number of measures and items that would increase the number of parameters tested and would require a large sample size, a path analysis was preferred over SEM. The path analysis was performed with MPLUS 8.11 software. The goodness of fit of the proposed model with the data was evaluated through incremental and absolute fit indices. In particular, χ^2^ was used as the main incremental index. Due to the fact that χ^2^ is heavily influenced by sample size, absolute fit indices were also used. More specifically, the Comparative Fit Index (CFI), the Root Mean Squared Error of Approximation (RMSEA) and the Standardized Root Mean Squared residual (SRMR) were used to estimate the fit of the model to the data. Based on existing recommendations, CFI scores close or above 0.95 were considered as demonstrating adequate fit. In addition, RMSEA and SRMR scores lower than 0.08 were considered as indicators of satisfactory model fit [53].

## 3. Results

Descriptive statistics and analysis of correlation are presented in Table 1. Correlation analyses revealed systematic patterns consistent with the proposed framework. Intentions to engage in CAPE behaviors were strongly associated with attitudes (r = 0.68, *p* < 0.001) and proximal subjective norms (r = 0.50, *p* < 0.001), and negatively associated with situational temptation (r = −0.47, *p* < 0.001). Intentions were also correlated with the means of performance enhancement (r = 0.40, *p* < 0.001), the combined means of training/work/study with nutritional supplements (r = 0.30, *p* < 0.001), and the combined means of training/work/study with PES (r = 0.35, *p* < 0.001).

At the distal level, attitudes correlated with knowledge (r = 0.20, *p* < 0.001), past behavior (r = 0.38, *p* < 0.001), distal subjective norms (r = 0.34, *p* < 0.001), distal descriptive norms (r = 0.28, *p* < 0.001), and autonomous motivation (r = 0.17, *p* < 0.001), and were negatively associated with control motivation (r = −0.17, *p* < 0.001). Proximal subjective norms showed strong correlations with knowledge (r = 0.24, *p* < 0.001), past behavior (r = 0.47, *p* < 0.001), motivation to comply (r = 0.62, *p* < 0.001), distal subjective norms (r = 0.41, *p* < 0.0001), distal descriptive norms (r = 0.48, *p* < 0.001), and autonomous motivation (r = 0.19, *p* < 0.001). Proximal descriptive norms were associated with knowledge (r = 0.34, *p* < 0.001), past behavior (r = 0.21, *p* < 0.001), motivation to comply (r = 0.35, *p* < 0.001), distal subjective norms (r = 0.17, *p* < 0.001), and distal descriptive norms (r = 0.39, *p* < 0.001).

Situational temptation was negatively associated with past behavior (r = −0.22, *p* < 0.001) and motivation to comply (r = −0.27, *p* < 0.001), and positively associated with control motivation (r = 0.19, *p* < 0.001). PBC was correlated only with distal subjective norms (r = 0.18, *p* < 0.001).

Finally, goal commitment was positively associated with the combined means of training/work/study and nutritional supplements (r = 0.25, *p* < 0.001) and the combined means of training/work/study and PES (r = 0.35, *p* < 0.001), and negatively associated with the means of performance enhancement (r = −0.38, *p* < 0.001).

The results of the path analysis showed an adequate fit model with the data [χ^2^ (129) = 204.953, *p* = 0.000; CFI = 0.941; SRMR = 0.050; RMSEA = 0.054, RMSEA CI90 lower limit = 0.040, RMSEA CI90 upper limit = 0.068].

With respect to the first hypothesis, results showed that the first distal subjective norms item (acceptance of CAPE by the general public) (β = 0.170, *p* < 0.05), proximal subjective norms (β = 0.544, *p* < 0.01) and past behavior (β = 0.168, *p* < 0.05) were positively associated with attitudes. Group identification, knowledge, cultural values, perfectionism and proximal descriptive norms were not associated with attitudes. With respect to perceived behavioral control, the results indicated that it was not associated with none of the studied variables (perfectionism, controlling and autonomous motivation, means of using PES and means of combining work/study/training and PES and past behavior). Controlling motivation (β = 0.166, *p* < 0.05) and past behavior (β = −0.201, *p* < 0.01) were positively and negatively associated with situational temptation, respectively, while perfectionism had a marginally insignificant negative association (β = −0.130, *p* = 0.052). Autonomous motivation was not significantly associated with situational temptation. With respect to proximal subjective norms, it was found to be associated with group identification (β = −0.112, *p* < 0.05), group orientation (β = −0.118, *p* < 0.05) and the second distal descriptive norms item (β = −0.130, *p* < 0.05). Motivation to comply (β = 0.392, *p* = 0.000), the first distal subjective norms item (acceptance of CAPE by the general public) (β = 0.206, *p* = 0.000), the second distal descriptive norms item (use by professionals in other work environments/students from other departments/athletes from other sports/exercisers in other exercise settings) (β = 0.189, *p* < 0.01) and past behavior (β = 0.251, *p* = 0.000) were positively associated with proximal subjective norms. Finally, group identification (β = 0.158, *p* < 0.05), motivation to comply (β = 0.168, *p* < 0.05), both distal descriptive norms (β = 0.184, *p* < 0.015 and β = 0.172, *p* < 0.05) and past behavior were positively associated with proximal descriptive norms, whereas cultural values were negatively associated (β = −0.131, *p* < 0.05). In contrast, group orientation and both distal subjective norms did not show significant associations with proximal descriptive norms (Table 2).

Regarding the second hypothesis that the proximal correlates of CAPE behaviors would be associated with intentions towards adopting CAPE behaviors and means, the results indicated that attitudes (β = 0.497, *p* = 0.000) and past behavior (β = 0.192, *p* = 0.001) were positively associated with intention to use PES, while situational temptation was negatively associated with intention to use PES (β = −0.224, *p* = 0.000). Perceived behavioral control, proximal subjective norms, proximal descriptive norms, goal commitment and goal difficulty were not significantly associated with intentions. With respect to the means of training/working/studying, the results indicated a negative association with proximal subjective norms (β = −0.244, *p* < 0.01), while attitudes, situational temptation, perceived behavioral control, and proximal descriptive norm, were not significantly related with these means. Regarding the means of using nutritional supplements, results revealed they were negatively associated with proximal subjective norms (β = −0.255, *p* = 0.010), while a positive association was found with descriptive norms (β = 0.263, *p* < 0.01). Attitudes, temptation and perceived behavioral control were not significantly related with the means of using nutritional supplements. Lastly, attitudes, temptation, perceived behavioral control, subjective norms and descriptive norms were not significantly related with the means of using substances, the means of combining training/work/study and use of nutritional supplements, the means of combining training/work/study and use of PES, as well as means of combining PES and nutritional supplement use (Table 3).

Concerning the third hypothesis that goal commitment and goal difficulty will be associated with means of performance enhancement, the results showed that goal difficulty was not significantly associated with any of the means (training/working/studying, using nutritional supplements, using substances, combining training/work/study and use of nutritional supplements, combining training/work/study and use of PES and combining PES and nutritional supplement use), while goal commitment (β = 0.258, *p* = 0.000) was positively associated with only the means of training/work/study.

With respect to the fourth hypothesis, it was found that PES as a means (β = 0.208, *p* < 0.05), was positively associated with intention to use PES. On the contrary, all other means, were not significantly related to intentions.

## 4. Discussion

The present study investigated a common conceptual framework for the understanding of CAPE behaviors in four different life domains. The results of the path analysis partially supported our hypotheses. Attitudes and situational temptation were associated with intentions to adopt CAPE behaviors, whereas perceived behavioral control and norms were not. Goal commitment was associated with the mean of working/studying/training but no other means. In addition, norms were associated with the means of using nutritional supplements. With respect to the associations between distal variables and proximal correlates of intentions, distal norms and past behavior were associated with attitudes, controlling motivation and past behavior was associated with situational temptation. Furthermore, cultural values, group identification, group orientation and distal descriptive norms, motivation to comply distal subjective norms and past behavior were associated with proximal norms.

The results of the study confirmed the association of distal variables with proximal correlates of intentions. Importantly, attitudes, social norms and situational temptation were the proximal correlates that were mostly associated with the distal variables. This finding highlights the important role these variables can play in the decision-making process [32,33]. The normative and cultural environment was most strongly related to these proximal variables. This finding signifies that CAPE behaviors are closely associated with the extent to which such behaviors are perceived as acceptable. In this case, a competitive mentality in achieving tangible outcomes and a broader mentality of improvement at all costs are linked to a dopogenic environment that increases the chances for CAPE behaviors. This was further corroborated by the observed association with controlling motivation, a type of motivation focusing on achieving rewards [42].

Attitudes and situational temptation emerged showed the strongest associations with CAPE behaviors intentions. This is in line with previous research [32,33,54] suggesting them as important variables in the decision-making process. These findings signify that individuals expecting positive outcomes from use or hold a positive view about substance use form stronger intentions towards use. Similarly, individuals who cannot resist the temptation to use these substances in order to achieve their goals have also higher intentions from use. Interestingly, perceived behavioral control that had been reported as important mediator in this process [32,33] was not significantly related to distal variables. This finding highlights an important trans-contextual mechanism. That is, perceived behavioral control may appears more relevant in competitive sport where the availability of prohibited substances and the potential sanctions are salients factors shaping the decision to use them. However, this may not be a barrier in work and studentship where PES are easily accessible and there are no restrictions in use. Accordingly, norms did not show significant associations, in contrast to previous evidence [55]. In this line, norms may be an important barrier for doping in sport due to the fact that it is considered unethical behavior [56]. However, in other life domains, such as exercise, work or studentship there may be no ethical concerns about the use of PES, and thus, norms may be less strongly related to substance use.

An important addition to the existing models of CAPE behaviors was the integration of aspects of the Goal System Theory [43]. More specifically we investigated the associations between proximal correlates of CAPE behavior on the means that individuals may use to achieve their objectives as well as the associations between these means and the intention for future CAPE behaviors. The results of the analysis indicated that using PES as means to achieve was positively associated with stronger intentions. This finding signifies a maladaptive reasoning suggesting that individuals considering PES use as a potential way to achieve are more susceptible in using such behaviors in the future. This evidence corroborates Barkoukis et al. [57] who advocated that a maladaptive mentality and biased reasoning results in favorable intentions towards CAPE behaviors. Interestingly, a mixed pattern was found on the association of norms with means. The means of working/studying/training was positively associated with subjective norms and negatively by descriptive norms. In addition, the means of using nutritional supplements was negatively associated with subjective norms and positively with descriptive norms. No clear explanations can be provided for these mixed findings. Clearly, more research is warranted to understand how norms relate to beliefs about effective means and reasoning for their use.

In interpreting these findings, it is important to acknowledge the substantial variability observed across participants. As shown in the descriptive data (Table 1), several variables, including intentions, temptation, and distal descriptive norms, displayed wide standard deviations, indicating that CAPE-related beliefs and tendencies were far from homogeneous. This heterogeneity carries theoretical significance, suggesting that individuals may differ not only in the strength but also in the configuration of their motivational and normative profiles. For example, some participants may experience strong temptation but weak intentions, whereas others may hold favorable attitudes or perceive permissive norms without necessarily engaging in use. Recognizing these distinct constellations of CAPE-related cognitions highlights the potential for person-centered approaches, such as cluster or latent-profile analyses, that could identify meaningful subgroups and refine the current framework by accounting for individual differences in vulnerability and goal regulation.

The study is not free of limitations. Firstly, its cross-sectional design and reliance on self-reported data preclude any causal inferences. Consequently, all findings describe associations rather than effects. In this respect, alternative explanations should be acknowledged. For instance, individuals with stronger CAPE intentions or previous experience with performance enhancement may retrospectively adjust their attitudes and perceptions of norms to justify their behavior. Likewise, cultural and social influences may not only shape but also be shaped by individual engagement in CAPE-related activities. To evolve into a fully testable theory, future research should generate and examine falsifiable hypotheses regarding the proposed causal pathways and their potential boundary conditions. Evidence inconsistent with these predictions, such as the reverse influence of intentions on attitudes or norms, would help refine or challenge the framework, advancing it from a conceptual to a predictive model. Future studies could employ longitudinal designs to establish the temporal ordering of variables and clarify potential feedback loops among distal, proximal, and behavioral components. Experimental or intervention-based approaches could further examine directionality by manipulating specific variables (e.g., attitudes, temptation, or normative perceptions) to test their effects on CAPE intentions or behaviors. Additionally, qualitative approaches could help unpack contextual meanings, providing insight into how individuals interpret sociocultural influences, motivational conflicts, and goal hierarchies underlying CAPE decisions. Also, the analytic approach presents limitations. The use of path analysis is ambitious given the modest overall sample size and the subgroup splits across four life domains. These conditions may increase the risk of overfitting and lead to unstable parameter estimates. Moreover, the use of path analysis without error estimation further limits the robustness of the conclusions. Future studies with larger and more balanced samples should employ structural equation modeling (SEM) to validate the model, account for measurement error, and confirm the stability of the observed associations. In addition, the small sample size in each life domain did not allow meaningful group comparisons. Future studies should include larger samples in each life domain and investigate via multigroup analyses the common and different paths across the domains. Lastly, our operationalization of CAPE encompassed substances of very different legal status and risk profiles. While dietary supplements and over-the-counter products are legally accessible, the use of anabolic steroids constitutes a controlled and more hazardous behavior. Future studies should examine these categories separately to disentangle common from substance-specific predictors.

## 5. Conclusions

The study findings provide preliminary evidence of a new conceptualization of CAPE behavior. Distal sociocultural and motivational variables are associated with proximal cognitive correlates of intentions and various means toward achieving higher-order goals. This is the first study to provide a conceptual framework and identify common correlates of CAPE behavior intentions across different life domains. In addition, it extends previous conceptualizations of CAPE behaviors by acknowledging that intentions are also influenced by the means that are available and that these means may guide behavior towards the attainment of a higher goal. The findings of the present study can provide the basis for the development of a common theoretical understanding of CAPE behavior (see Figure 1) and accordingly develop effective interventions to help people achieve informed decisions about the ways they can use to achieve their goals. The present findings carry several practical implications for educational, occupational, and sport settings. Understanding the sociocultural and motivational factors associated with CAPE behaviors can inform the development of prevention and education programs that address both individual and contextual influences. In applied contexts, coaches, educators, and health professionals can play a pivotal role in shaping ethical norms and promoting non-chemical pathways to achievement by encouraging self-regulation, adaptive goal pursuit, and critical reflection on social pressures for performance. Interventions based on this framework may emphasize informed decision-making and psychological well-being rather than performance outcomes alone.

Taken together, the findings indicate that a set of factors, namely motivation to comply, group identification, distal descriptive and subjective norms, and past behavior, play a key role in shaping the proximal cognitive processes associated with CAPE. Through their influence on attitudes, proximal norms, and situational temptation, these variables contribute to the formation of CAPE intentions. In addition, goal commitment and the use of PES as a means further strengthen this pathway. Overall, these significant factors form an empirically grounded model that can meaningfully enhance our understanding of how CAPE behaviors develop.

## Figures and Tables

**Table 1:** Descriptive statistics of the study variables.

	Mean	Standard Deviation	Cronbach α
Intention	2.08	1.36	0.89
Mean work	6.40	1.02	-
Mean supplements	3.08	1.63	-
Mean PES	1.97	1.51	-
Mean work and supplements	3.23	2.26	-
Mean work and PES	2.67	2.06	-
Mean supplements and PES	1.97	1.57	-
Goal commitment	5.92	1.06	-
Goal difficulty	4.95	1.53	-
Attitudes	2.08	1.17	0.86
Temptation	5.37	1.83	0.87
Perceived behavioral control	5.46	1.30	-
Proximal subjective norms	2.05	1.08	0.77
Proximal descriptive norms	2.11	1.59	-
Group identification	4.29	1.26	0.67
Group orientation	5.63	1.12	-
Knowledge	1.82	1.19	0.63
Cultural values	3.86	0.68	0.73
Perfectionism	4.01	0.67	0.67
Past behavior	1.20	0.56	-
Motivation to comply	2.81	1.06	-
Distal subjective norms 1	3.17	1.75	-
Distal subjective norms 2	2.78	2.01	-
Distal descriptive norms 1	3.53	1.43	-
Distal descriptive norms 2	43.88	26.33	-
Control motivation	6.00	1.15	0.83
Autonomous motivation	3.82	1.17	0.84

**Table 2:** Standardized associations between distal and proximal variables related to CAPE behaviors (H1).

Outcome	Predictor	β	*p*
Attitudes	Distal subjective norm 1 (acceptance by general public)	0.170	<0.05
Attitudes	Proximal subjective norms	0.544	<0.01
Attitudes	Past behavior	0.168	<0.05
Attitudes	Group identification, knowledge, cultural values, perfectionism, proximal descriptive norms	ns	>0.05
PBC	Perfectionism, controlling/autonomous motivation, mean PES, mean combined work/study/training + PES, past behavior	ns	>0.05
Situational temptation	Controlling motivation	0.166	<0.05
Situational temptation	Past behavior	−0.201	<0.01
Situational temptation	Perfectionism	−0.130	0.052
Situational temptation	Autonomous motivation	ns	>0.05
Proximal subjective norms	Group identification	−0.112	<0.05
Proximal subjective norms	Group orientation	−0.118	<0.05
Proximal subjective norms	Distal descriptive norm 2	−0.130	<0.05
Proximal subjective norms	Motivation to comply	0.392	0.000
Proximal subjective norms	Distal subjective norm 1	0.206	0.000
Proximal subjective norms	Distal descriptive norm 2	0.189	<0.01
Proximal subjective norms	Past behavior	0.251	0.000
Proximal descriptive norms	Group identification	0.158	<0.05
Proximal descriptive norms	Motivation to comply	0.168	<0.05
Proximal descriptive norms	Distal descriptive norm 1	0.184	<0.015
Proximal descriptive norms	Distal descriptive norm 2	0.172	<0.05
Proximal descriptive norms	Cultural values	−0.131	<0.05
Proximal descriptive norms	Group orientation, distal subjective norms	ns	>0.05

Note. β = standardized regression coefficient; PBC = perceived behavioral control; ns = non-significant.

**Table 3:** Standardized scores of proximal correlates of intentions and means of CAPE behaviors (H2).

Outcome	Predictor	β	*p*
Intention to use PES	Attitudes	0.497	0.000
Intention to use PES	Past behavior	0.192	0.001
Intention to use PES	Situational temptation	−0.224	0.000
Intention to use PES	PBC, prox. subjective norms, prox. descriptive norms, goal commitment, goal difficulty	ns	>0.05
Mean: Work/study/train	Proximal subjective norms	−0.244	<0.01
Mean: Nutritional supplements	Proximal subjective norms	−0.255	0.010
Mean: Nutritional supplements	Descriptive norms	0.263	<0.01
Mean: Substances; combined means (work/study/training + supplements or PES)	All predictors	ns	>0.05

Note. β = standardized regression coefficient; ns = non-significant.

## Data Availability

The data presented in this study are not publicly available due to privacy and ethical restrictions related to participant confidentiality. Data may be made available upon request from the corresponding author.

## Abbreviations

The following abbreviations are used in this manuscript:

CAPE	Chemically Assisted Performance Enhancement
TPB	Theory of Planned Behavior
IMBP	Integrative Model of Behavioral Prediction
PES	Performance Enhancement Substances
CFI	Comparative Fit Index
RMSEA	Root Mean Squared Error of Approximation
SRMR	Standardized Root Mean Squared
